# Seasonal Changes in the Tetrodotoxin Content of the Flatworm *Planocera multitentaculata*

**DOI:** 10.3390/md15030056

**Published:** 2017-02-25

**Authors:** Riko Yamada, Tadasuke Tsunashima, Mitsuki Takei, Tatsunori Sato, Yoshiki Wajima, Makoto Kawase, Shotaro Oshikiri, Yusuke Kajitani, Keita Kosoba, Hiroyuki Ueda, Koko Abe, Shiro Itoi, Haruo Sugita

**Affiliations:** 1 Department of Marine Science and Resources, Nihon University, Fujisawa, Kanagawa 252-0880, Japan; rikopin.0202@gmail.com (R.Y.); t007_moyasi@yahoo.co.jp (T.T.); mitsuki.takei.nubs@gmail.com (M.T.); tatsunorisato.nubs@gmail.com (T.S.); wajimayoshi.nu@gmail.com (Y.W.); brma13039@g.nihon-u.ac.jp (M.K.); n.u.oshikirishotaro@gmail.com (S.O.); himajin0090@gmail.com (Y.K.); ueda.h.65039@gmail.com (H.U.); abe.kouko@nihon-u.ac.jp (K.A.); sugita.haruo@nihon-u.ac.jp (H.S.); 2 The Graduate School of Marine Science and Technology, Tokyo University of Marine Science and Technology, Minato, Tokyo 108-8477, Japan; keita.kosoba@gmail.com

**Keywords:** flatworm, planocerid, *Planocera multitentaculata*, pufferfish, tetrodotoxin (TTX)

## Abstract

Tetrodotoxin (TTX) is a potent neurotoxin that acts specifically on voltage-gated sodium channels on excitable membranes of muscle and nerve tissues. The biosynthetic process for TTX is unclear, although marine bacteria are generally thought to be the primary producers. The marine flatworm *Planocera multitentaculata* is a known TTX-bearing organism, and is suspected to be a TTX supplier to pufferfish. In this study, flatworm specimens were collected from an intertidal zone in Hayama, Kanagawa, Japan, the TTX content of the flatworm was measured using liquid chromatography with tandem mass spectrometry (LC-MS/MS), and seasonal changes in TTX content were investigated. No significant difference in TTX concentration of the flatworm body was found between the spawning period and other periods. However, the TTX content in individual flatworms was significantly higher in the spawning period than at other times. The TTX content rose in association with an increase in the body weight of the flatworm.

## 1. Introduction

Tetrodotoxin (TTX, C_11_H_17_N_3_O_8_), known as pufferfish toxin, is a sodium channel blocker, and is one of the most toxic natural substances known [1,2,3]. The fatal dose of tetrodotoxin to humans (50 kg body weight) is only 1–2 mg [4]. TTX has been detected from taxonomically diverse organisms across 14 different phyla, including pufferfish and amphibians [5,6], fish [7], cephalopods [8], gastropods [9,10,11], bivalves [12], crustaceans [13], starfishes [14,15], flatworms [16,17,18,19] and ribbonworms [17]. Several of the species that carry TTX have been found to be prey organisms of pufferfish [20]. TTX production has also been observed in several species of bacteria that are symbiotic with the pufferfish and their potential food organisms [21,22,23]. Therefore, accumulation of TTX in pufferfish is thought to be due to biomagnification through the food chain, starting with marine bacteria as the primary TTX producer [3,23]. Interestingly, TTX is not detected in artificially cultured pufferfish fed on non-toxic organisms after hatching, although cultured non-toxic pufferfish can be induced to harbor TTX by oral administration of the toxin [24,25,26,27,28].

The ecology of TTX-bearing organisms at lower trophic levels (e.g., flatworms) had not been investigated except for some investigations [19,29]. Their classification is controversial and their life histories also remain unclear. There is evidence that flatworms are involved in the accumulation of TTX in pufferfish. Cultured Japanese pufferfish (*Takifugu rubripes*) fed on the toxin bearing the polyclad flatworm *Stylochus orientalis*, which can migrate into aquaculture ponds on land, showed increased levels of TTX [29]. Additionally, flatworms have been reported to contribute to the accumulation of toxins in other animals: dog neurotoxicosis has been shown to result from the consumption of the side-gilled sea slug (*Pleurobranchaea maculate*) in coastal areas of New Zealand [10], and subsequent investigation revealed that the sea slugs contained the toxin as a consequence of their predation of the polyclad flatworm *Stylochoplana* sp. [11]. Marine polyclad flatworms are common in coastal and rocky reefs worldwide [30], and they play influential roles in marine ecosystems [31,32,33,34]. The flatworm *Planocera multitentaculata*, which is a potential prey species of pufferfish around the Japanese coastal area [16,35], has been reported to accumulate relatively high levels of TTX. This has led to the suggestion that *P. multitentaculata* is important when considering the cycle of TTX among toxic organisms (the so-called TTX loop), particularly as many of the links in this cycle are still unknown [36].

Many of the pufferfish *Takifugu niphobles*, one of highly toxic species, and the toxic flatworm *P. multitentaculata* sympatrically inhabit the coastal/intertidal zones around the Miura Peninsula, Japan, and the toxin amounts in the pufferfish around the waters were extremely high (maximum TTX content: females 1.9 mg/individual and males 6.5 mg/individual [37]). Therefore, in this study, we investigated seasonal changes in the whole body total toxin content and concentration of TTX in the flatworm *P. multitentaculata*. Our analyses have revealed a new aspect of the ecology of this species, and our data will be of value to elucidating the contribution of this flatworm to toxin accumulation in pufferfish.

## 2. Results

### 2.1. Seasonal Changes in Body Weight and Sexual Maturity of the Flatworm

**Figure 1:**
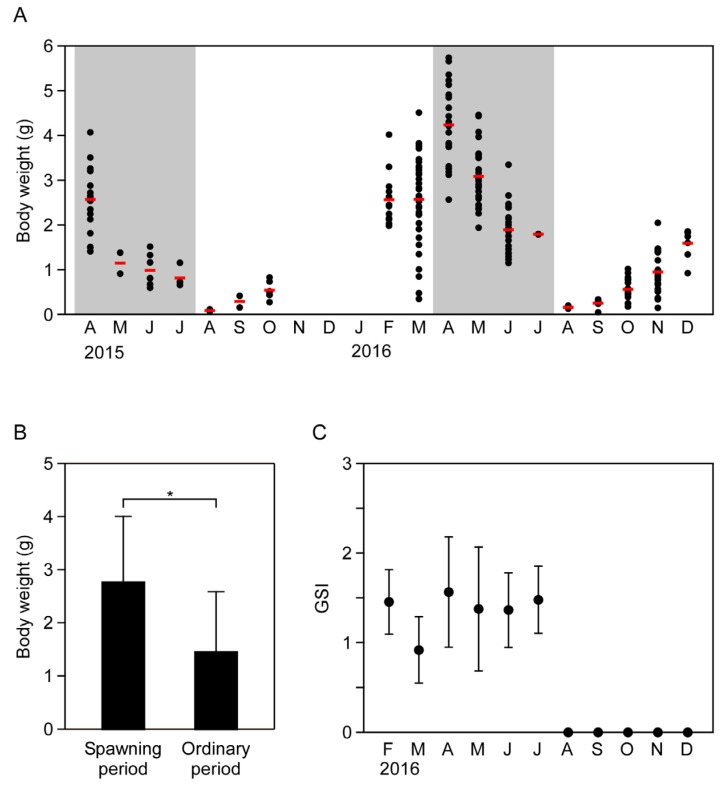
Seasonal changes in body weight and gonadosomatic index (GSI) of the flatworm *Planocera multitentaculata*. (**A**) Seasonal changes in the body weight. Circles and red bars represent individual and mean data, respectively; (**B**) Differences in body weight between spawning period and other times of the year. Student’s *t*-test was used for the analyses (* significant at *p* < 0.05); (**C**) Seasonal changes in GSI. Data are means ± standard deviation.

Seasonal changes in flatworm body weights are shown in Figure 1. Body weight began to increase in September, peaked in April (mean ± SD: 4.24 ± 0.86 g), and then gradually decreased from May to August (Figure 1A). Mean body weight was significantly higher in the spawning period (April to July) than at other times of the year (*p* < 0.05, Figure 1B). Seasonal changes in the gonadosomatic index (GSI) are shown in Figure 1C. The mean GSI from February to July ranged from 0.91 to 1.48; gonadal tissues were not present from August to December. Sexually mature individuals were observed only between April and July, and adults and egg plates were observed under rocks in the intertidal zone during this period. Benthic young and adult flatworms were present in relatively low numbers in August and also had the lowest average body weights (*n* = 2, 0.09 g in 2015; *n* = 2, 0.17 g in 2016).

### 2.2. Seasonal Changes in Toxin Concentration and Total Toxin Content in the Flatworms

**Figure 2:**
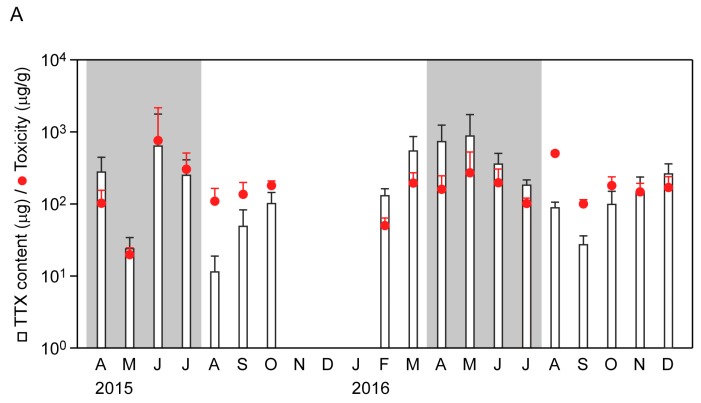

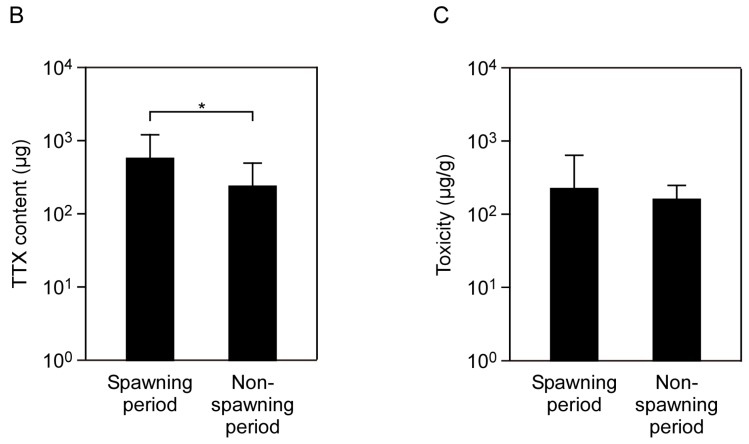
Seasonal changes in TTX content/toxicity of the flatworm *Planocera multitentaculata*. (**A**) Seasonal changes in TTX content and toxicity of the whole body. TTX content and toxicity are represented by column and circle, respectively. White and gray zones indicate ‘non-spawning period’ and ‘spawning period’, respectively. Data are monthly means + standard deviation (SD); (**B**) Comparison of the toxicity of flatworms in spawning and non-spawning periods. Data are means + SD; (**C**) Difference in TTX contents in spawning and non-spawning periods. Data are means + SD. Student’s *t*-test was employed for statistical analysis (* significant at *p* < 0.05).

**Figure 3:**
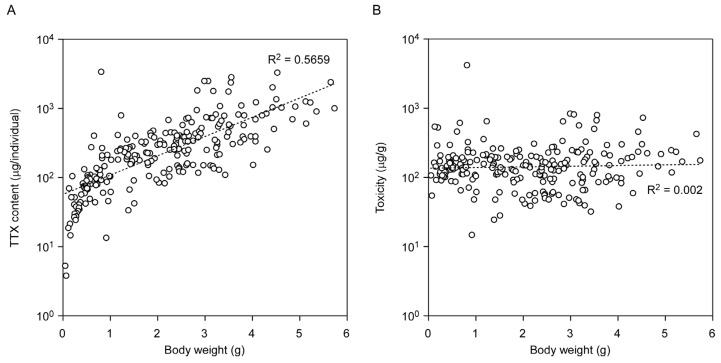
Effect of body weight on the amount of TTX in the whole body of the flatworm *Planocera multitentaculata*. Panel (**A**) shows the relationship between TTX content and body weight; panel (**B**) shows relationship between toxicity and body weight.

LC-MS/MS was used to obtain a mass chromatogram under the multiple-reaction monitoring (MRM) mode, which showed that the MRM chromatograms obtained from the flatworm body extracts exhibited similar chromatographic retention characteristics to those observed following the analysis of TTX standard (Figure S1). A calibration curve generated using 1–100 ng/mL TTX standards showed good linearity and precision (*y* = 106.548*x* + 14.515, *R*^2^ = 0.9975). As shown in Figure 2, the mean whole body TTX content exceeded 11 μg/individual, which corresponded to approximately 50 MU/individual (see Materials and Methods for description of the toxicity assessment). No individuals were found that lacked the toxin. A maximum TTX content of 3388 μg/individual (corresponding to approximately 15,400 MU/individual) was found in June and the maximum mean ± SD of 819 ± 874 μg/individual (corresponding to 3723 ± 3973 MU/individual) was found in May. The total amount of TTX per flatworm decreased during the spawning period in 2016, although toxicity was relatively constant. On the other hand, data showed variability in the total TTX trend within the spawning period in 2015. The mean of the TTX concentrations during the present study ranged from 101 ± 23 μg/g to 757 ± 1516 μg/g, except for those in two months (20 μg/g in May 2015; 51 μg/g in February 2016). Student’s *t*-tests of the whole-body TTX contents showed that seasonal variations were significant (*p* < 0.05), whereas toxicity did not show significant changes (Table S1). Whole-body TTX content was related to body weight to some extent (y = 57.168e^0.6409x^, *R*^2^ = 0.5659), whereas the TTX concentration was not (y = 135.85e^0.0229x^, *R*^2^ = 0.002; Figure 3). There is very wide variability among animals in TTX amount.

### 2.3. Tissue Localization of TTX in the Flatworm

**Figure 4:**
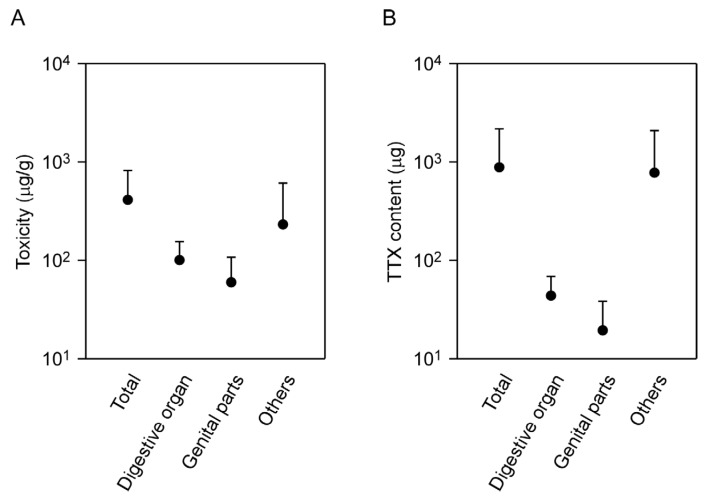
TTX distribution in the body of the flatworm *Planocera multitentaculata*. (**A**) Comparison of toxicity of different tissues from the flatworms; (**B**) Difference in TTX contents of different tissues. “Total” represents the whole body including digestive organ, genital parts and remaining tissue. Data are means + standard deviation.

We investigated the distribution of TTX in the flatworm body by dissection of five individuals collected in April 2015 (Figure S2). No significant differences were observed in the TTX concentrations of the digestive organ (102 ± 52 μg/g), genital parts (61 ± 46 μg/g) and remaining tissue (236 ± 374 μg/g) (Figure 4). The mean amount of toxin in the remaining tissues (817 ± 1333 μg) was markedly higher than that in the digestive organ (46 ± 24 μg) and genital parts (20 ± 19 μg), but no significant difference was present.

### 2.4. Toxicity of Eggs and Larvae

**Figure 5:**
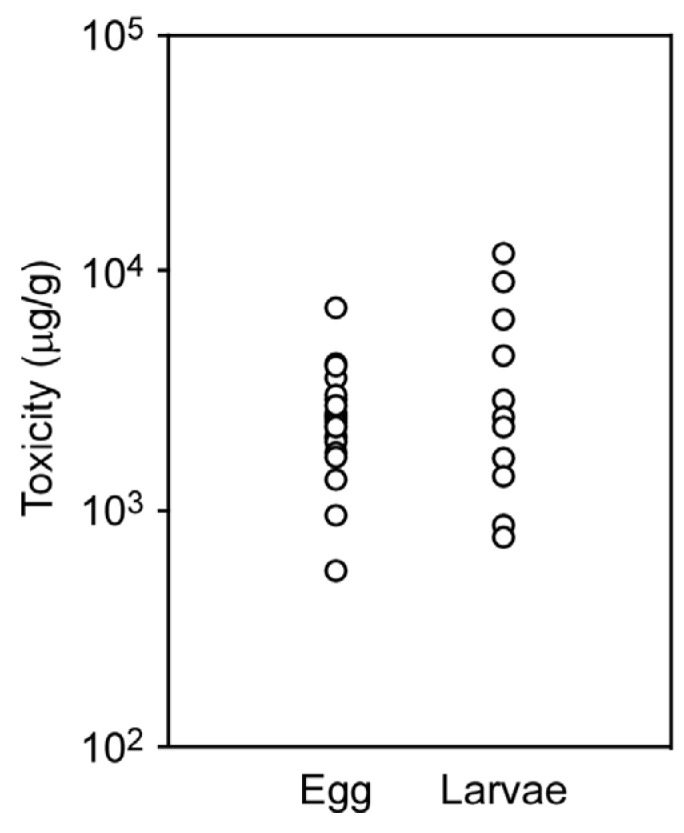
Toxicity of egg plates and larvae of the flatworm *Planocera multitentaculata*. Twenty-two egg plates and 12 batches of larvae were subjected to LC-MS/MS analysis.

Flatworms raised in the laboratory deposited egg plates on the aquarium wall during the spawning season (April to July). The egg plates were collected and incubated, and hatched larvae were later collected. Analysis of the TTX concentrations of the egg plate and larvae yielded 2563 ± 1332 μg/g and 4042 ± 3500 μg/g, respectively (Figure 5); these values are comparable to those of adult flatworms. The mean level of toxin in larvae was estimated at 58.7 ± 27.7 ng/individual, and that of the egg plate could not be estimated because of seasonal/within-population variability in the size/weight of the egg plate.

## 3. Discussion

Our analysis of TTX levels in the whole flatworm body throughout the year showed that these varied significantly among seasons. TTX contents were significantly higher in the spawning period than at other times of the year; however, this effect was not found for the TTX concentration. In parallel, the GSIs of the flatworm were higher in the spawning period, including before the two months, than at other times: the high SD of the GSI suggests that there was a difference in the sexual maturation process and number of spawning times among the flatworm individuals, because the flatworm spawns many times within a season. *Planocera multitentaculata* was the first polyclad organism to be reported as being TTX-bearing [16]; this initial description was followed by reports of other toxic flatworm species including *P. reticulata* and *Stylochus ijimai* [38].

In the present study, we show that the TTX contents in *P. multitentaculata* showed seasonal variation, with a peak in the spawning period. The latter peak is associated with an increase in body weight. By contrast, TTX concentrations did not differ throughout the year. Several studies have suggested that planocerid flatworms invest a high level of TTX in their progeny as a defense against predators [35,39]. Another possibility was reported in an unidentified planocerid flatworm that utilized TTX both for defense and for catching prey [18]. In pufferfish, TTX might be provided to larvae for defense against predators and also released to act as an aggregation pheromone during the spawning period [36,37,40,41,42]. Further investigations are required to elucidate the full gamut of ecological and physiological functions of TTX in flatworms and other species.

**Figure 6:**
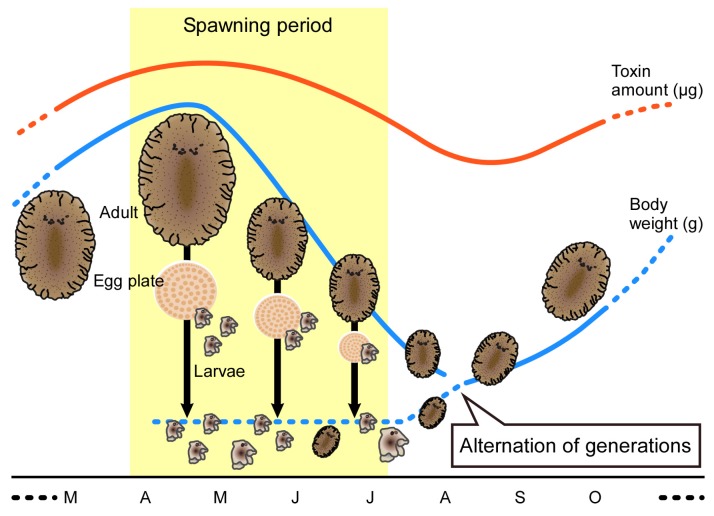
Schema showing the life history and changes in TTX content of the flatworm *Planocera multitentaculata*. The flatworm lays eggs underneath stones during the spawning period. The body weight/size decreased in association with time during the spawning period. Recruitment of the flatworm was sequentially observed during the spawning period (April–July). Hatched larvae settled and individuals of the new generation were found in August–September. These newly settled individuals had a high level of TTX.

Our analyses demonstrated that the mean body weight of the flatworms increased from September to a peak in April, and then decreased from May to August. Some individuals of small size were collected in August and September. These population dynamics suggest that the alternation of generations occurs in the summer (Figure 6). If this suggestion of alternation of generations is correct, it raises the interesting question of how the flatworms accumulate large amounts of TTX. Salvitti et al. [43] reported that no TTX-producing bacteria could be isolated from the intestinal contents of the flatworm *Stylochoplana* sp. On the other hand, Ritson-Williams et al. [18] suggested that TTX and its analogues in an undescribed planocerid species might be produced either endogenously or by symbiotic bacteria. Speculation on the role of symbiotic bacteria in TTX production has been supported by the isolation of *Vibrio* sp., *Vibrio alginolyticus*, *Shewanella alga*, and *Alteromonas tetraodonis* from various TTX-bearing animals including the pufferfish *Takifugu vermicularis vermicularis*, a xanthid crab *Atergatis floridus*, starfish *Astropecten polyacanthus* and the red calcareous alga *Jania* sp. [21,44,45,46]. Nevertheless, the amount of TTX produced by bacteria is unknown, but it is unlikely to be sufficient to explain the level of TTX in the flatworm body. The mechanisms for accumulating the toxin in the flatworm are key to the cycle of TTX among toxic organisms.

Organisms carrying TTX can show various population structures [47]: (1) TTX and non-TTX-containing populations/individuals are found; (2) TTX concentrations are extremely variable within populations; (3) no relationship is observed between body/tissue weight and the TTX concentration; (4) seasonal differences in TTX concentrations are marked within populations; (5) concentrations of TTX are different among organs/tissues; (6) TTX is passed to the progeny. Our analysis of *P. multitentaculata* shows that characteristics 1, 3 and 5 did not apply. In the case of *P. multitentaculata*, a high concentration of TTX was detected in all individuals in this study. Additionally, the TTX content rose with the increase in body weight. We did not find evidence of any significant differences in TTX concentrations among the genitalia, digestive organ, and remaining tissues. These patterns of TTX distribution suggest that the TTX concentration of TTX-bearing organisms in the area might depend on the biomass of the flatworm. Further studies will be required to determine whether this speculation is correct.

## 4. Materials and Methods

### 4.1. Flatworm Individuals

The flatworms were collected at the fixed point by turning the stones within a compartment of 10 m square in an intertidal zone in Hayama, Kanagawa, Japan (35°15′ N, 139°34′ E, Figure S3) from April 2015 to December 2016. Flatworm larvae were obtained from egg plates spawned in laboratory aquaria. All samples were stored at −20 °C until TTX extraction.

### 4.2. Assessment of the Gonadosomatic Index (GSI)

The GSI of each flatworm was calculated from its gonadal area (GA) and body area (BA) (Figure S4) using Canvas 12 software (ACD Systems of America, Inc., Fort Lauderdale, FL, USA) and the following equation:

GSI = (GA/BA) × 100

### 4.3. LC-MS/MS Analysis

As described in our previous report [37], TTX was extracted from samples using 0.1% acetic acid. The extract was then filtered through a 0.45 μm filter membrane (SupraPure Syringe Filter, PTEE-Hydrophilic, Recenttec, Taipei, Taiwan) and was subjected to analysis by liquid chromatography with tandem mass spectrometry (LC-MS/MS). Quantification of the TTX was performed using a Quattro Premier XE (Waters, Milford, MA, USA) equipped with an electrospray ionization (ESI) source coupled to an Acquity UPLC system (Waters) [36]. Chromatographic separation was achieved using an Atlantis HILIC Silica column (2.1 × 150 mm, 5 μm; Waters), coupled to an Atlantis HILIC Silica pre-column (2.1 × 10 mm, 5 μm; Waters), with gradient elution using formic acid/acetonitrile. The mass spectrometer was operated in MRM mode, with detection in positive mode, and analysis of two product ions at *m*/*z* 162 for quantification of TTX and *m*/*z* 302 for confirmation of the compound from the precursor ion at *m*/*z* 320. A calibration curve was generated using 1 to 100 ng/mL of a TTX standard (Wako Pure Chemicals, Osaka, Japan); this showed good linearity and precision. Quantification of TTX was carried out using the data for samples with >1000-fold dilution to remove influence of matrix effects, because TTX was recovered from the samples with >1000-fold dilution. One mouse unit (MU) was defined as the amount of toxin required to kill a 20 g male ddY strain mouse within 30 min after intraperitoneal administration, and equivalent to 0.22 µg of TTX, based on the specific toxicity of TTX [48].

### 4.4. Statistical Analysis

Between-group data in the spawning period and the rest of the year were analyzed by Student’s *t*-test. Tukey-Kramer post-hoc tests were used to analyze the data on TTX concentrations among genitalia, digestive organ, and remaining tissues. Student’s *t*-tests and Tukey-Kramer post-hoc tests were performed using Microsoft Excel and the R v. 3.1.1 software [49], respectively. A significance level of *p* < 0.05 was set.

## 5. Conclusions

In summary, the seasonal changes in TTX contents of the flatworm *P. multitentaculata* depended on body weight and not on changes in TTX concentrations. TTX amounts per individual were significantly higher in the spawning period than at other times of the year. No significant differences in TTX concentration were observed between the spawning period and other times of the year. Seasonal changes in body size of the flatworm population suggested that the alternation of generations occurred in the summer.

## Supplementary Materials

The following are available online at http://www.mdpi.com/1660-3397/15/3/56/s1, Figure S1: Typical mass chromatograms from LC-MS/MS (MRM mode, *m*/*z* 320 > 302) of whole bodies of the flatworm *Planocera multitentaculata*, Figure S2: Schematic dissection of the flatworm *Planocera multitentaculata* and distribution of TTX in different body tissues, Figure S3: Sampling locality for the flatworm *Planocera multitentaculata*, Figure S4: Dorsal and ventral views of the flatworm *Planocera multitentaculata*, Table S1: *Planocera multitentaculata* individuals used in this study and estimation of their toxicity levels and TTX amounts.
