# Supplementary material for: Seasonal Changes in the Tetrodotoxin Content of the Flatworm Planocera multitentaculata
Source: Mar Drugs. 2017 Feb 25;15(3):56. doi: 10.3390/md15030056 (PMC5367013; doi:10.3390/md15030056)
Supplement: Supplementary file 1 [file marinedrugs-15-00056-s001.pdf]

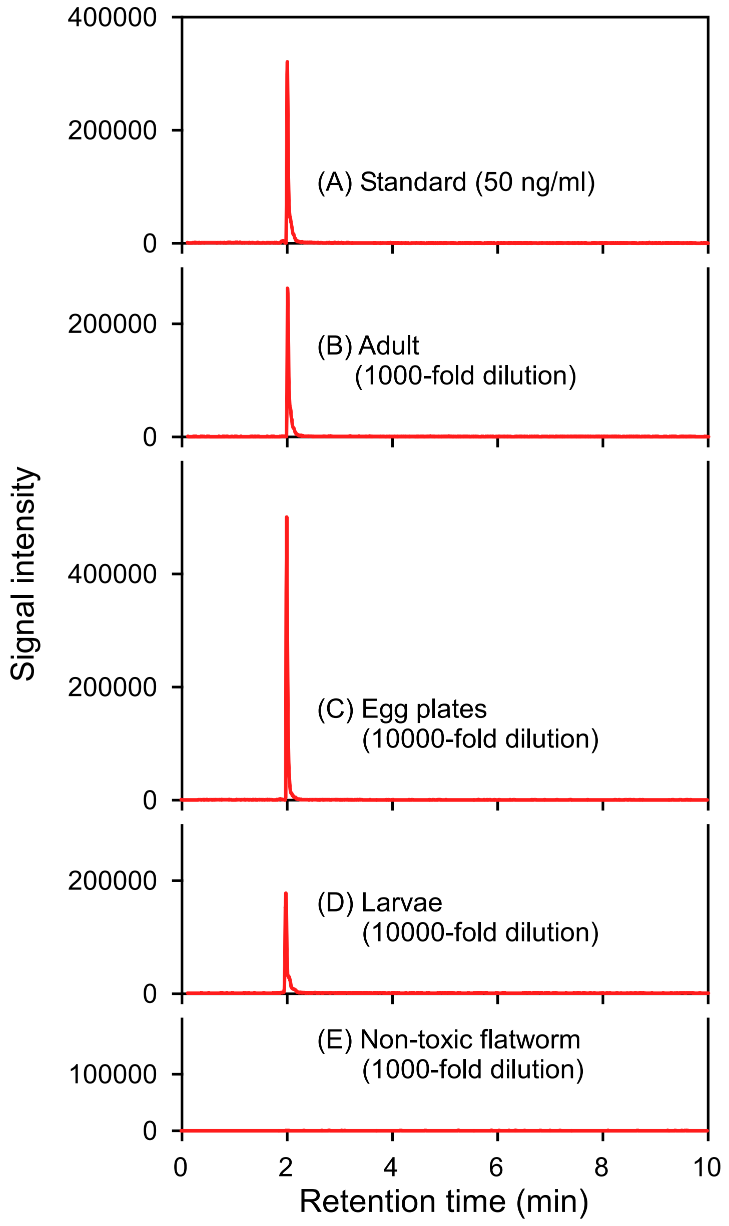

Figure S1. Typical mass chromatograms from LC-MS/MS (MRM mode, *m*/*z* 320 > 302) of whole bodies of the flatworm *Planocera multitentaculata*. MRM patterns of 50 ng/ml TTX standard (A), extracts from adult flatworms (B), extracts from egg plates (C), extracts from larvae (D) and extract from the non-toxic flatworm *Discoplana gigas* (E).

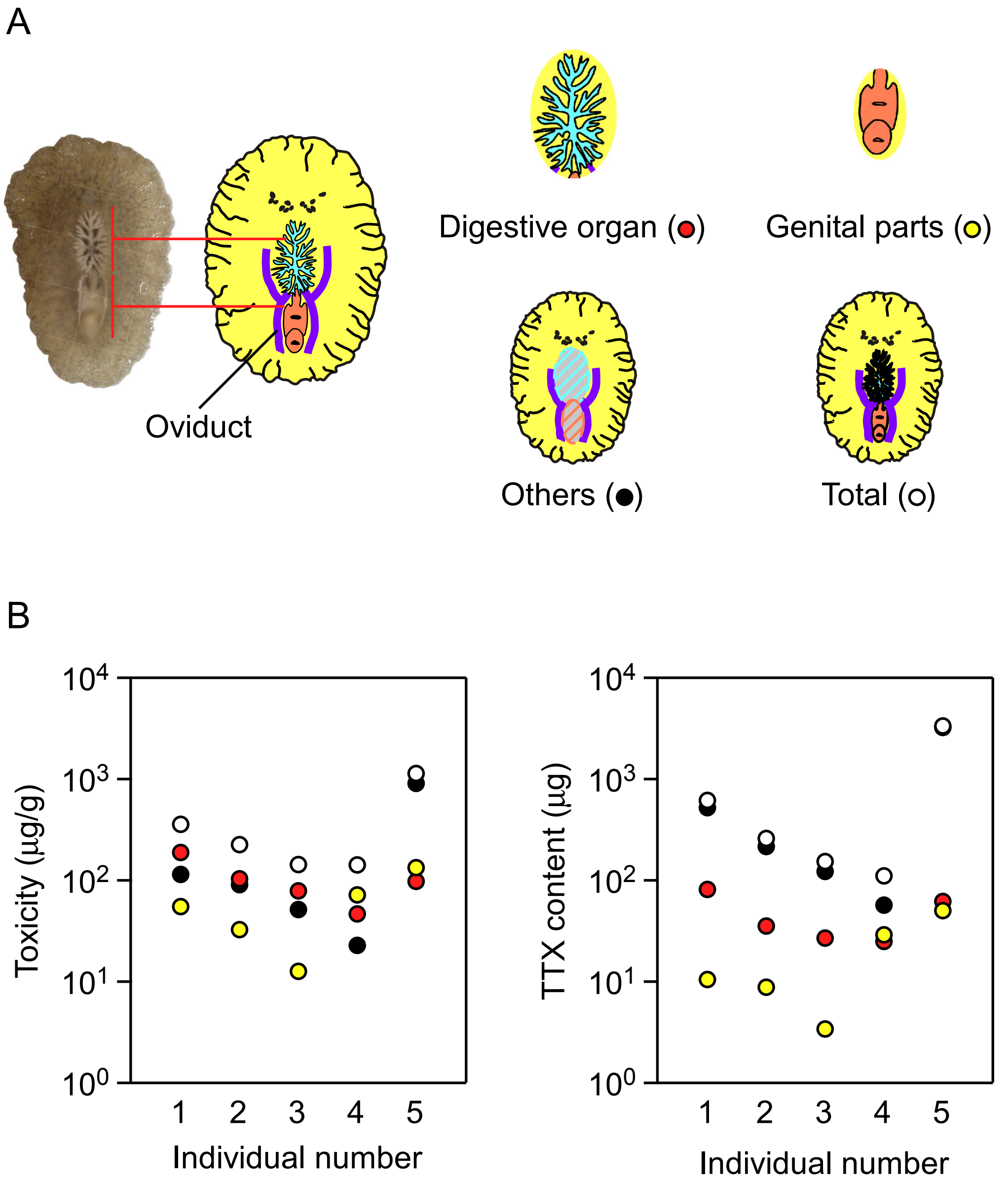

Figure S2. Schematic dissection of the flatworm *Planocera multitentaculata* and distribution of TTX in different body tissues. A, Flatworms were dissected into digestive organs, genitalia and remaining tissues. B, Toxicity (left panel) and TTX content (right panel) of tissues from five individuals. Red, yellow and black circles represent toxicity/TTX contents of digestive organ, genitalia and remaining tissues, respectively, whereas white circles represents TTX content of the whole body.

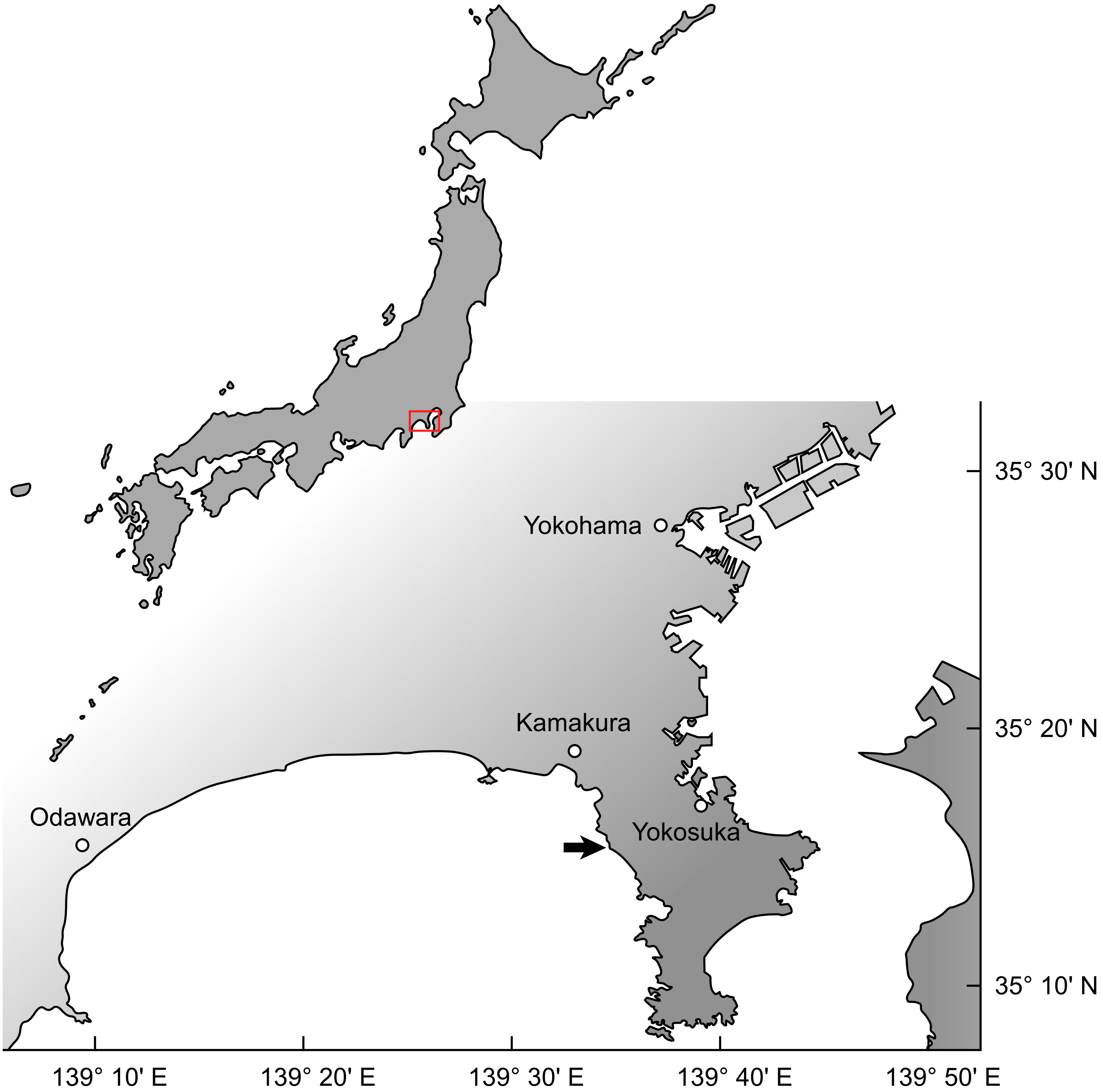

Figure S3. Sampling locality for the flatworm *Planocera multitentaculata*. Arrow in the map indicates the sampling site used in this study.

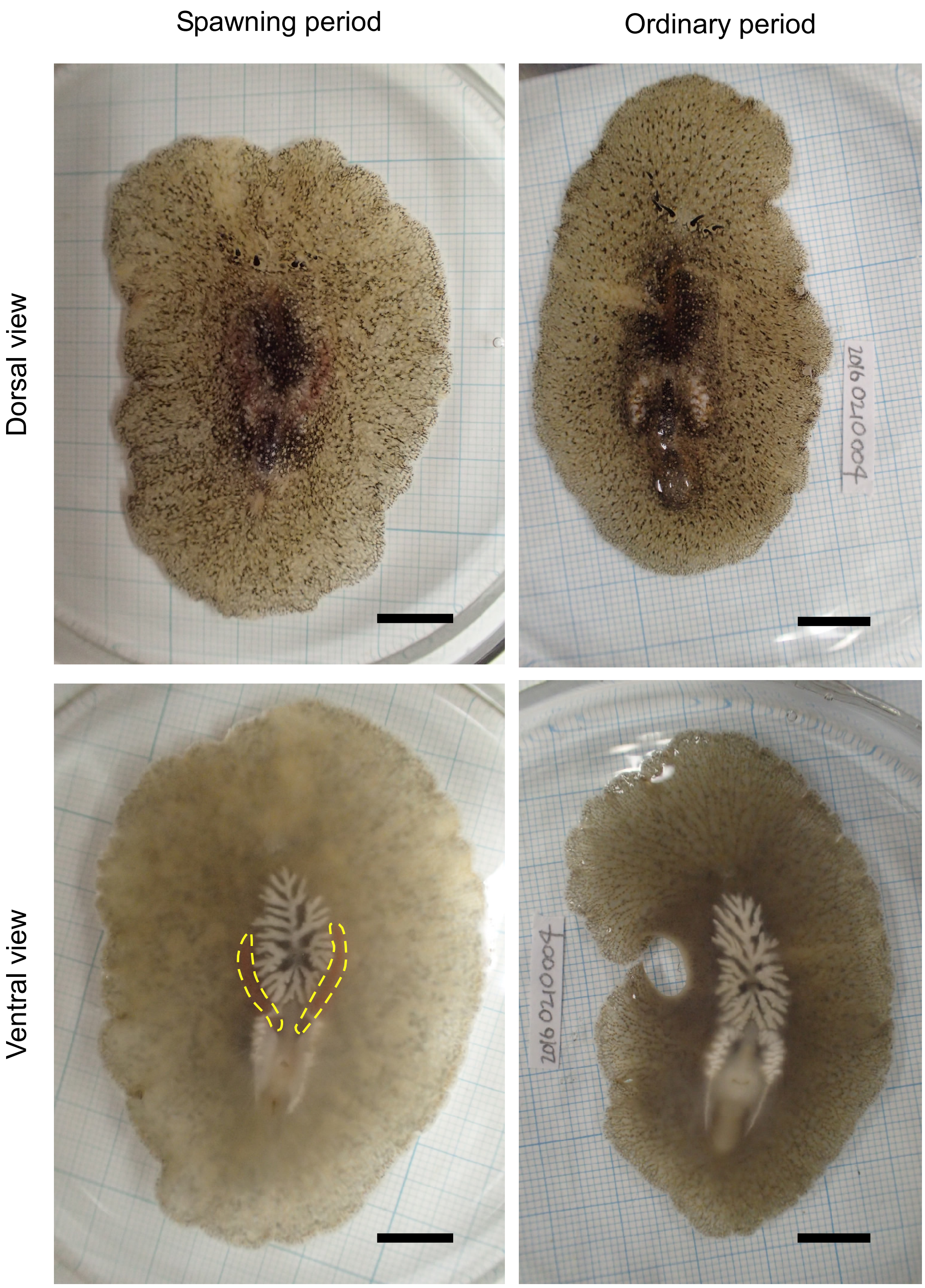

Figure S4. Dorsal and ventral views of the flatworm *Planocera multitentaculata*. Left and right panels show flatworms during spawning and non-spawning periods, respectively. Upper and lower panels show dorsal and ventral views of flatworms, respectively. The gonadal areas of flatworms in the spawning period are indicated by yellow dotted lines. Bars, 10 mm.

Table S1. *Planocera multitentaculata* individuals used in this study and estimation of their toxicity levels and TTX amounts [a]

Collection month		No. of individuals	Body weight (g)	Toxicity (μg/g)	TTX amount (μg/individual)
2015	April	18	2.57 ± 0.71	102 ± 52	275 ± 167
	May	2	1.15 ± 0.23	20 ± 5	24 ± 10
	June	7	0.99 ± 0.33	757 ± 1404	634 ± 1127
	July	4	0.82 ± 0.20	303 ± 204	248 ± 161
	August	2	0.09 ± 0.02	109 ± 54	11 ± 7
	September	2	0.29 ± 0.13	144 ± 52	49 ± 34
	October	8	0.54 ± 0.16	179 ± 28	101 ± 43
2016	February	13	2.56 ± 0.53	51 ± 12	129 ± 33
	March	35	2.57 ± 0.95	195 ± 75	543 ± 318
	April	22	4.24 ± 0.86	159 ± 85	730 ± 508
	May	29	3.09 ± 0.64	270 ± 255	874 ± 862
	June	21	1.89 ± 0.54	197 ± 106	357 ± 146
	July	3	1.79 ± 0.01	101 ± 18	182 ± 33
	August	2	0.17 ± 0.04	533 ± 7	88 ± 17
	September	8	0.26 ± 0.08	106 ± 8	27 ± 9
	October	20	0.56 ± 0.22	179 ± 58	98 ± 51
	November	22	0.95 ± 0.46	146 ± 47	144 ± 91
	December	7	1.61 ± 0.34	170 ± 70	261 ± 101

[a] Data are presented as means ± standard deviation.
